# Identification of M2-like macrophage-related signature for predicting the prognosis, ecosystem and immunotherapy response in hepatocellular carcinoma

**DOI:** 10.1371/journal.pone.0291645

**Published:** 2023-09-19

**Authors:** Qian Feng, Hongcheng Lu, Linquan Wu

**Affiliations:** 1 Department of Emergency, The Second Affiliated Hospital of Nanchang University, Nanchang, China; 2 Department of General Surgery, The Second Affiliated Hospital of Nanchang University, Nanchang, China; Abu Dhabi University, UNITED ARAB EMIRATES

## Abstract

**Background:**

Hepatocellular carcinoma is one of the most common malignancies worldwide, representing a big health-care challenge globally. M2-like macrophages are significantly correlated with tumor progression, metastasis and treatment resistance.

**Methods:**

Integrative 10 machine learning algorithms were performed to developed a M2-like macrophage related prognostic signature (MRPS). Single-cell RNA-sequencing analysis was performed to dissect the ecosystem of HCC. Several approaches, including TIDE score, immunophenoscore, TMB score and tumor escape score were used to evaluate the predictive role of MRPS in immunology response.

**Results:**

The optimal MRPS constructed by the combination of stepCox + superPC algorithm served as an independent risk factor and showed stable and powerful performances in predicting the overall survival rate of HCC patients with 2-, 3-, and 4-year AUCs of 0. 763, 0.751, and 0.699 in TCGA cohort. HCC patients with low risk score possessed a more interaction of immunoactivated cells, including NK, CD8^+^ cytotoxic T, and activated B, and a less interaction of immunosuppressive cells, including Treg, CD4^+^ exhauster T, and M2-like macrophage. Low risk score indicated a higher PD1&CTLA4 immunophenoscore, higher TMB score, lower TIDE score and lower tumor escape score in HCC, suggesting a better immunotherapy response. The IC50 value of docetaxel, gemcitabine, crizotinib and Osimertinib in HCC with high risk score were lower versus that with low risk score. HCC patients with high risk score had a higher score of cancer-related hallmarks, including angiogenesis, DNA repair, EMT, glycolysis, and NOTCH signaling.

**Conclusion:**

Our study proposed a novel MRPS for predicting the prognosis, ecosystem and immunotherapy response in HCC.

## 1. Introduction

Hepatocellular carcinoma is one of the most common malignancies worldwide, representing a big health-care challenge globally [1]. Every year, an estimated of 905,677 million cases are diagnosed with HCC and 830,180 patients died of this disease globally [2]. In the USA, the average 5-year survival rate of HCC patients is 19.6% but can be as low as 2.5% in patients with advanced disease ormetastatic disease [3]. High invasiveness, recurrence and metastasis are the main reasons affecting the prognosis of HCC patients [4]. HCC is usually diagnosed at advanced stages, making the tumor unresectable and the treatments limited [3]. Resistance to chemotherapy, targeted therapy and immunotherapy are the main reason for treatment failure [5]. Considering the heterogeneity of the HCC, constant searching at molecular level for screening biomarker using for stratifying the ecosystem, the prognosis and drug sensitivity of HCC patients is gaining greater importance.

Tumor microenvironment (TME) is composed of cancer cells, stromal cells, immune cells, cytokines, chemokines and so on [6]. The complex crosstalk between HCC and TME could ultimately reprogram an environment favoring tumor growth and metastasis [7]. Tumor associated macrophages (TAMs) could be divided into two M1 phenotype and M2 phenotype [8]. Most of macrophages were M1 phenotype in the tumor early development while the function phenotype of macrophages slowly changes into M2 phenotype with tumor progression, leading to immune suppression and tumor angiogenesis [9,10]. TAMs constitute a plastic and heterogeneous cell population of TME, accounting for about 50% of some solid neoplasm [11]. Increasing evidences revealed that M2-like TAMs were significantly correlated with tumor progression, metastasis and treatment resistance [12,13]. Thus, comprehensively studying the characteristics of M2-like TAMs -related genes (MRGs) may elucidate their correlation with the prognosis, ecosystem, therapeutic response of HCC patients.

Single-cell RNA sequencing (scRNA-seq) has suggested as a good approach for clarifying the molecular characteristics and ecosystem of cancer [14]. Integrated analysis of single-cell and bulk RNA-sequencing bring hug potential for identifying molecular biomarker and mechanisms associated with the prognosis and immunotherapy of cancer [15]. Herein, we developed a M2-like TAMs -related prognostic signature (MRPS) for HCC using bulk RNA-seq data. Moreover, we also clarified the correlation between MRPS and the ecosystem as well as drug sensitivity of HCC patients by integrated analysis of single-cell and bulk RNA-sequencing data. Our results may provide more evidences for the prognostic markers and therapeutic targets for HCC.

## 2. Materials and methods

### Data acquisition

**Fig 1:**
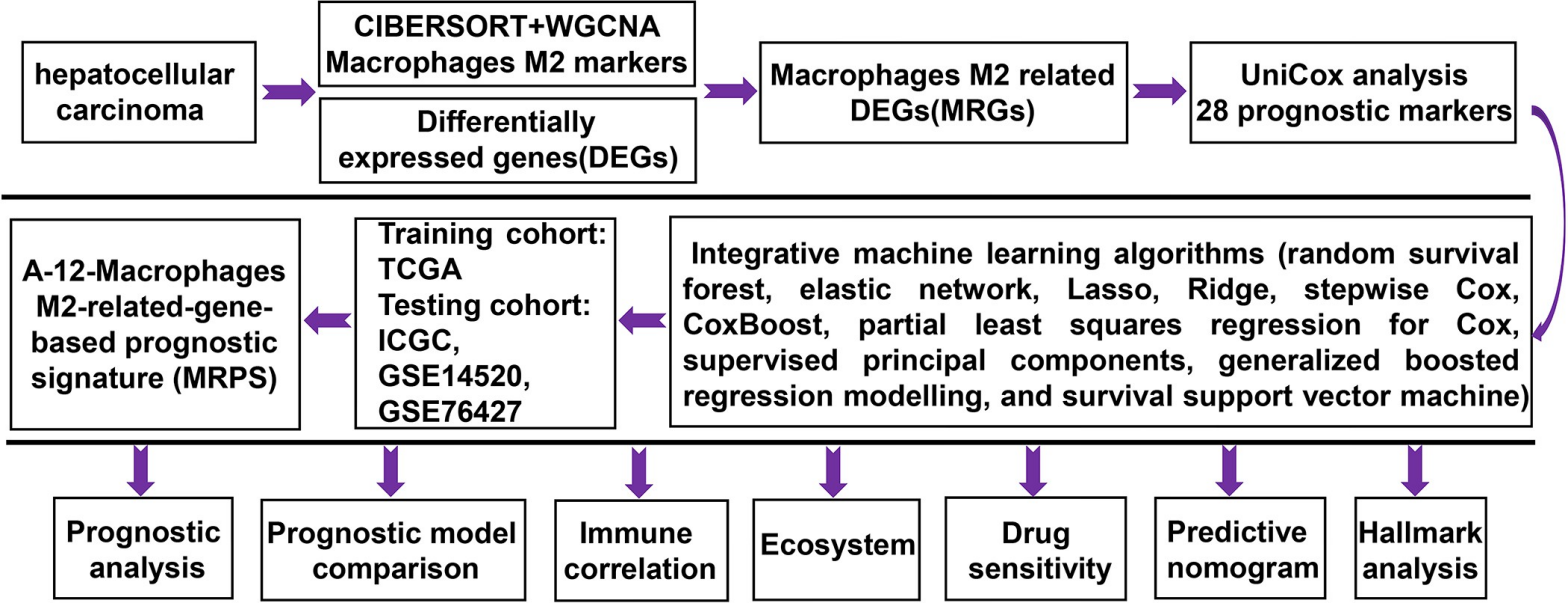
Study workflow of our study.

Flow chart of the current study was showed in Fig 1. From the GSE149614 dataset, we obtained the scRNA-seq data of HCC tumor samples (n = 10). For developing and verifying the prognostic signature, we collected four bulk RNA-seq datasets of HCC, including TCGA (n = 329), ICGC (n = 228), GSE14520 (n = 218) and GSE72094 (n = 90). Inclusion criteria were as following: (1) histologically diagnosed with HCC, (2) completed follow-up time over three months. Exclusion criteria were as following: (1) metastatic HCC. Two immunotherapy datasets (IMigor210 (n = 298) and GSE91061 (n = 89)) were using for evaluating the correlation between the MRPS-based risk score and immunotherapy response.

### CIBERSORT and WGCNA

CIBERSORT predicts the proportion of 22 immune cells in tumour sample expression data based on linear support vector regression principles. Bulk RNA-seq data of TCGA HCC dataset was summitted to CIBERSORT algorithm and generate the expression of immune cells [16]. weighted gene co-expression network analysis (WGCNA) is a reliable tool to identify gene sets of interest from thousands of the most varied genes and clarify correlation analysis with phenotypes. To obtain strong correlations between genes in the adjacency matrix, we set β value under the degree of independence of 0.9 in network construction. Those samples with a mean expression>0.5 were selected for analysis. Neighbourhood relationships were then converted into a topological overlap matrix (TOM) and clustered in a chain hierarchy based on the mean of different TOM-based metrics. Those genes in the module that displayed the significant positive correlation with macrophage M2 were defined as MRGs.

### Integrative machine learning algorithms constructed an optimal MRPS

DEGs among MRGs were determined by “limma” package using |LogFC| ≥ 1.5 as the cutoff. Univariate cox analysis was performed to identify prognostic biomarkers in HCC with p<0.05 as threshold value. To construct an accurate and stable prognostic MRPS for HCC, we then conducted integrative analysis with 10 machine learning algorithms, including random survival forest (RSF), elastic network (Enet), Lasso, Ridge, stepwise Cox, CoxBoost, partial least squares regression for Cox (plsRcox), supervised principal components (SuperPC), generalized boosted regression modelling (GBM), and survival support vector machine (survival-SVM). Prognostic biomarkers were firstly submitted to integrative machine learning procedures in the TCGA dataset, which could determine the candidate genes in MRPS and their corresponding coefficient within the leave-one-out cross-validation framework. The machine learning algorithms was followed as previous studies [17,18] and the link to the R scripts are available on the Github website (https://github.com/Zaoqu-Liu/IRLS). Based on the candidate genes and their corresponding coefficient, we then calculated the risk score and Harrell’s concordance index (C-index) of HCC cases in TCGA, ICGC, GSE14520, and GSE76427 cohort. The prognostic signature with the highest average C-index was regarded as the optimal MRPS.

### Assessment of MRPS

The best cut-off for distinguishing between high and low risk group was determined with the survminer R package.HCC patients were separated into high and low risk score groups in each cohorts using best cut-off. The survival curves of high and low risk score groups were generated using Kaplan-Meier survival method. ROC curve and C-index of MRPS were drawn using the survivalROC R package. By performing univariate and multivariate Cox analyses, we then determined the risk factors for the prognosis of HCC patients. After randomly collected 10 prognostic signatures that have been developed for HCC and calculated their C-index, we then compared the C-index of our prognostic signature with these 10 prognostic signatures. Considering MRPS-based risk score and other clinical parameters, we also constructed a predictive nomogram using nomogramEx package, which could predict the 1-, 3-, and 5-year OS rates of HCC samples. The difference between actual and predicted survival was visualized using the calibration curve. Calibration curves (by bootstrap method with 500 resamples) and receiver operating characteristic (ROC) curves were applied to evaluate the nomogram. A 45-degree line in the plot represents perfect calibration, where predicted probabilities match the actual outcomes. A decision curve analysis (DCA) curve was drawn using the rmda package to evaluate the possibility of MRPS for clinical application.

### Immune infiltration analysis

ESTIMATE analysis was performed to detect the immunoscore, stromascore and ESTIMATEScore of each HCC case [19]. Immunedeconv, an R package integrating six state-of-the-art algorithms including: TIMER, xCell, MCP-counter, CIBERSORT, EPIC, and quanTIseq was utilized to evaluate the abundance of immune cells of each HCC case [20]. Each algorithm was systematically benchmarked and found to have unique properties and strengths. The “ggpubr” or “ggplot” R package was used to visualize relative expression patterns of human leukocyte antigen (HLA)-related genes and immune checkpoints in HCC. The infiltration scores of 16 immune cells and the function of 13 immune-related pathways was determined by.single-sample gene set enrichment analysis (ssGSEA). The immune cells were as follows: activated dendritic cells (aDCs); B cells; CD8 T cells; DCs; immature DCs (iDCs); macrophages; mast cells; neutrophils; natural killer cells (NK cells); plasmacytoid DCs (pDCs); T helper cells; T follicular helper (Tfh) cells; Th1 cells; Th2 cells; tumor infiltrating lymphocytes (TILs); regulatory T cells (Tregs).

### scRNA-seq analysis

scRNA-seq data was processed with the Seurat R package (version 4.0) [21]. Those genes detected in fewer than 3 cells, cells with fewer than 200 detected genes or cells with a mitochondrial proportion of over 20% would not select for further analysis. The top 2000 highly variable genes of each sample were normalized using the ScaleData function based on variance stabilization transformation (vst). After normalizing gene expression using LogNormalize method, we made cell populations on a two-dimensional map by performing principal component analysis (PCA) and T-distributed Stochastic Neighbor Embedding (tSNE) analysis. Cell type annotation depend on the expression of the marker genes (S1 Table). AddModuleScore function was performed to calculate the scores of MRPS in each single cell in these 10 HCC samples. Based on the ligand-receptor information, we used the single-cell gene expression matrix to unravel the communication between different cell subtypes which was contained in CellChat software with default parameters, modelling the communication probability and identifying significant communications.

### Drug sensitivity analysis

Several primary scores, including the Tumor Immune Dysfunction and Exclusion (TIDE) score, immunophenoscore (IPS), Tumor Mutation Burden (TMB) score and tumor escape score, were apply to evaluate the role of MRPS in predicting the immunotherapy response of HCC cases. TIDE score of HCC patients were downloaded from TIDE (HTTP://tide.dfci.harvard.edu/). The immunophenoscore (IPS) of HCC patients was obtained from The Cancer Immunome Atlas (TCIA, https://tcia.at/home). Lower TIDE score, lower tumor escape score, higher IPS and higher TMB score indicate a less likelihood of immune escape and better effectiveness of ICI treatment. Another two immunotherapy cohorts, GSE91061 (n  =  39, anti-CTLA4 and anti-PD1 therapy) and IMigor210 (n  =  298, anti-PD1 therapy), were used to verify the role of the risk score in predicting the immunotherapy response. The IC50 of drugs in each HCC was evaluated with the oncoPredict R package based on the data of Genomics of Drug Sensitivity in Cancer (https://www.cancerrxgene.org/). The sensitivities of these drugs were reflected by comparing the IC50 values in patients with high- and low- risk score. A higher IC50 value indicated lower sensitivity.

### Statistical analysis

R version 4.1.3 and GraphPad Prism 9 were used to perform statistical studies. The difference between different survival curve was calculated by the log-rank test. Correlation coefficients were determined by Spearman analysis. The differences between two groups were evaluated by the Wilcoxon test. The proportional hazard assumption was assessed using the Schoenfeld individual test, and visually inspected for potential time-variant biases. Double-tailed P < 0.050 was considered statistically significant.

## 3. Results

### Identification of potential prognostic biomarker among MRGs in HCC

**Fig 2:**
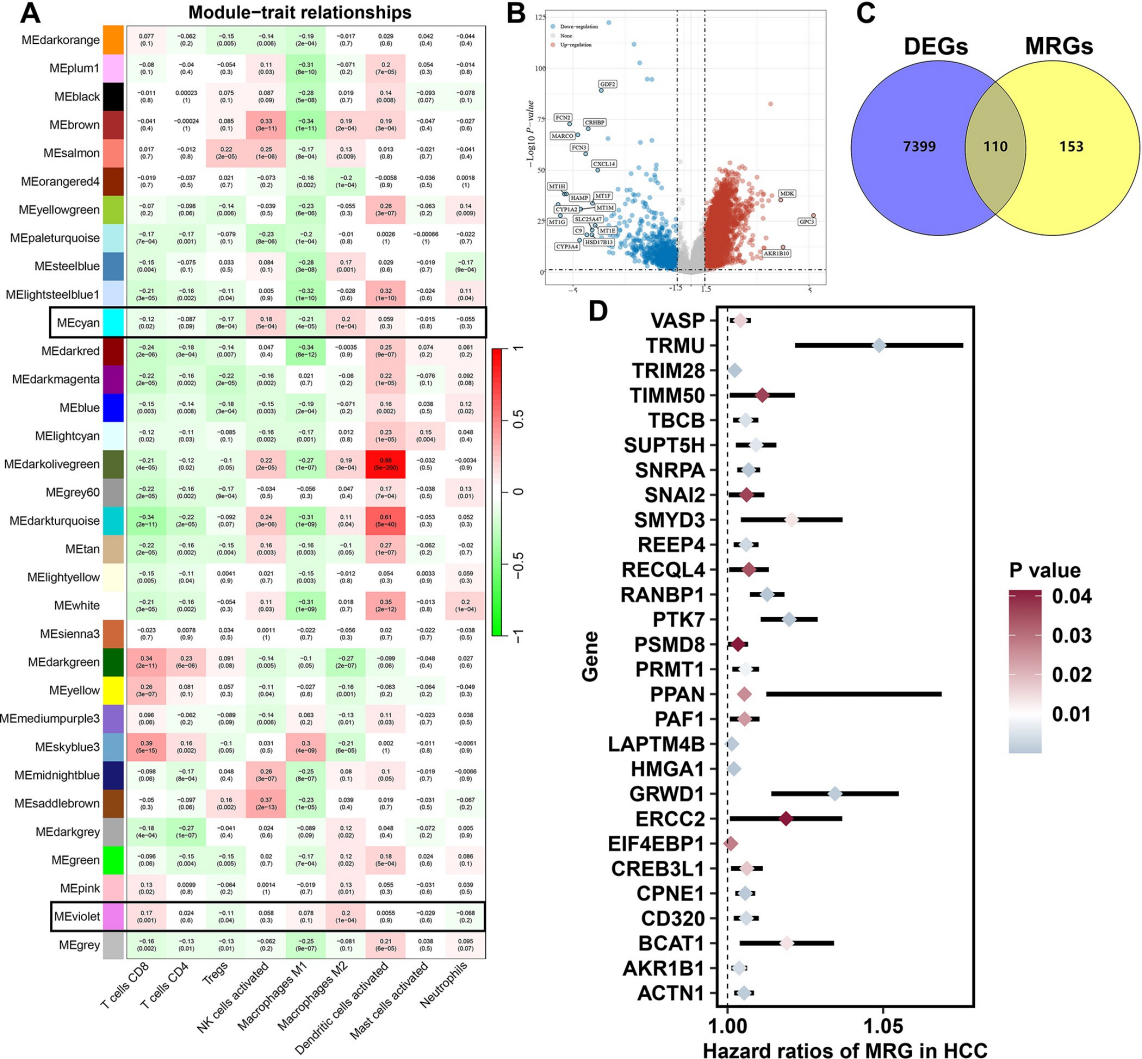
Identification of potential biomarkers of M2-like macrophage-related genes in HCC. (A) WGCNA identified M2-like macrophage related genes in HCC. (B) Heatmap showing the differentially expressed genes in HCC. (C) The overlap of M2-like macrophage related genes and differentially expressed genes in HCC. (D) The potential prognostic biomarkers of M2-like macrophage-related genes in HCC.

To identify M2-like MRGs in HCC, we performed WGCNA analysis. As shown in Fig 2A, the violet and cyan modules showed a highest positive correlation with macrophage M2 (cor = 0.2, *p* = 1e^-4^). The genes in the violet and cyan modules were defined as M2-like MRGs (S1 Table). With |LogFC| ≥ 1.5 as the threshold, we obtained 7509 DEGs in HCC (Fig 2B). Among these DEGs, a total of 110 genes were differentially expressed MRGs (Fig 2C, S2 Table). Further cox univariate analysis identified 28 differentially expressed MRGs were significantly correlated with the prognosis of HCC patients (Fig 2D, *p*<0.05).

### Integrative machine learning algorithms developed an optimal prognostic MRPS

**Fig 3:**
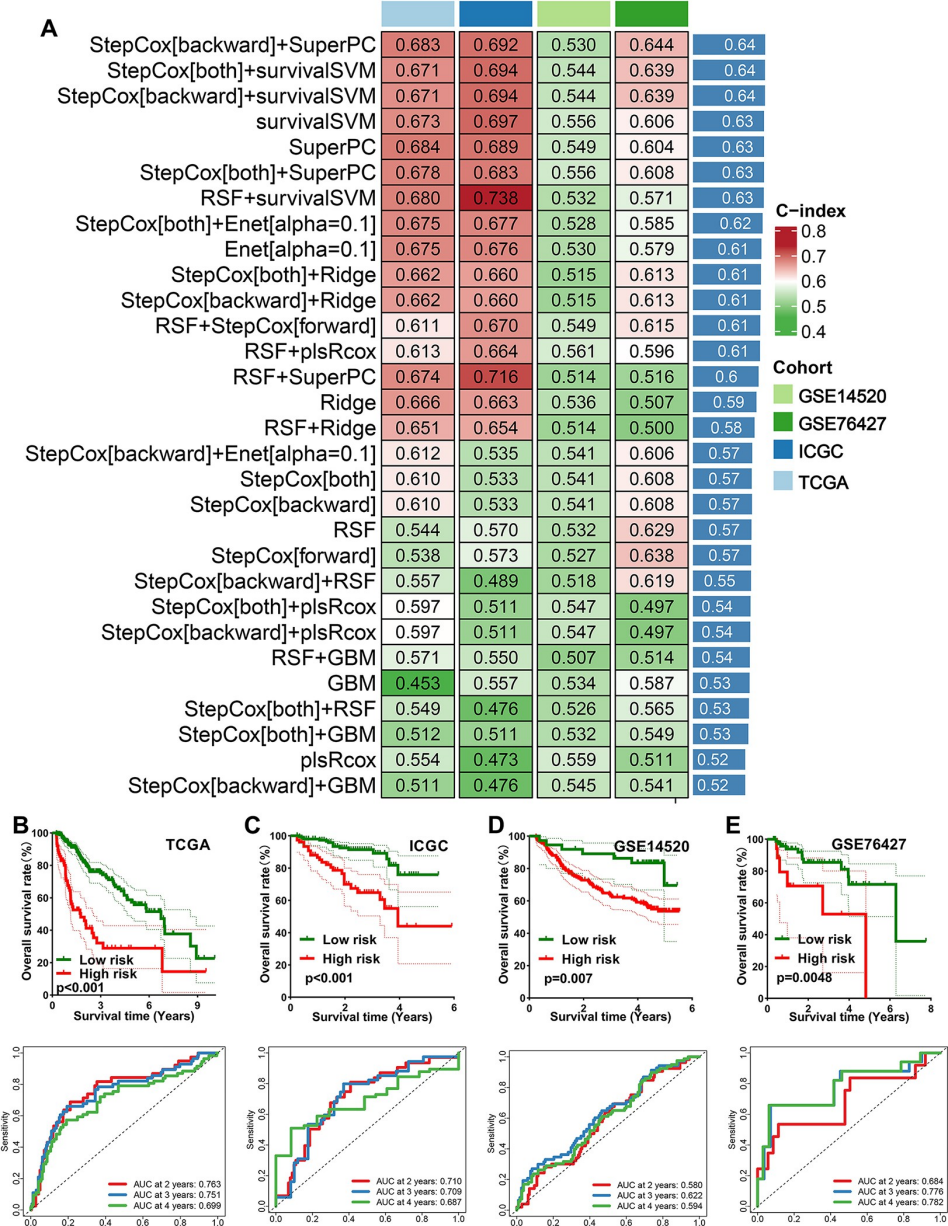
Identification and evaluation of a M2-like macrophages-related prognostic signature (MRPS). (A) The C-index of each prognostic model constructed by 10 machine learning algorithms in training and testing cohort. The survival curve and corresponding TimeROC curve of HCC with high and low risk score in TCGA (A), ICGC (B), GSE14520 (C) and GSE76427 (D) cohort.

Using 10 machine learning-based methods, we generated 30 kinds of prognostic models (Fig 3A). The C-index of each prognostic model in training cohort (TCGA) and testing cohort (ICGC, GSE14520, GSE76427) were shown in Fig 3A. As a result, the model constructed by StepCox[backward] + SuperPC method was considered as the optimal model and they had a highest average C-index of 0.64 (Fig 3A). This optimal MRPS was developed using 12 MRGs, and the risk score of each HCC case was calculated using following formula: risk score = 0.5671879 × SNRPA^exp^ + (-0.4282688) ×RECQL4^exp^ + 0.6410663 ×TRIM28^exp^ + (-0.3376426) × ERCC2^exp^ + 0.3590617 × GRWD1^exp^ + 0.4491523 × REEP4^exp^ + (-1.2190593) × PRMT1^exp^ + (-0.3341503) × PAF1^exp^ + 0.2864963 × LAPTM4B ^exp^ + 0.3805174 × RANBP1^exp^+ (-0.3052321) × BCAT1^exp^ + 0.4239179 × PTK7^exp^. HCC cases were divided into high and low risk groups using the best cut-off. As shown in Fig 3B–3E, HCC patients with high risk score had a poor OS rate in TCGA (*p*<0.001), ICGC (*p*<0.001), GSE14520 (*p* = 0.007), and GSE76427 cohort, with 2-, 3-, and 4-year AUCs of 0. 763, 0.751, and 0.699 in TCGA cohort; 0.710, 0.709, and 0.687 in ICGC cohort; 0.580, 0.622, and 0.594 in GSE14520 cohort; and 0.684, 0.776, and 0.784 in GSE76427 cohort, respectively.

### Evaluation of the performance of MRPS

**Fig 4:**
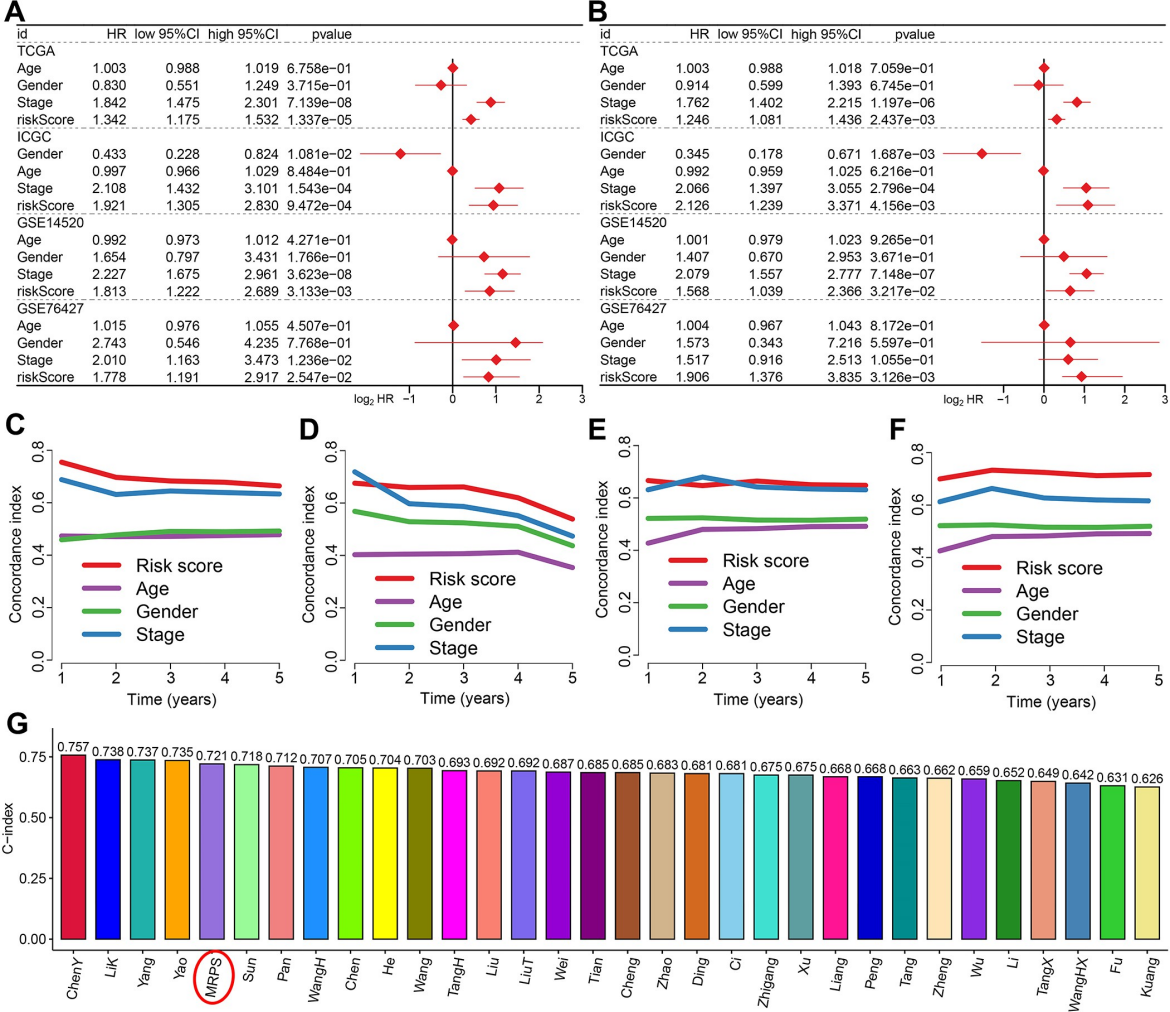
Evaluation of the performance of M2-like macrophages-related prognostic signature (MRPS). Univariate (A) and multivariate (B) cox regression analysis considering age, gender, stage and risk score in training and testing cohort. (C-F) C-index curve evaluated the discrimination of MRPS in predicting the overall survival rate Of HCC patients in training and testing cohort. (G) The C-index of MRPS and other 31 prognostic models that have been developed for HCC.

**Fig 5:**
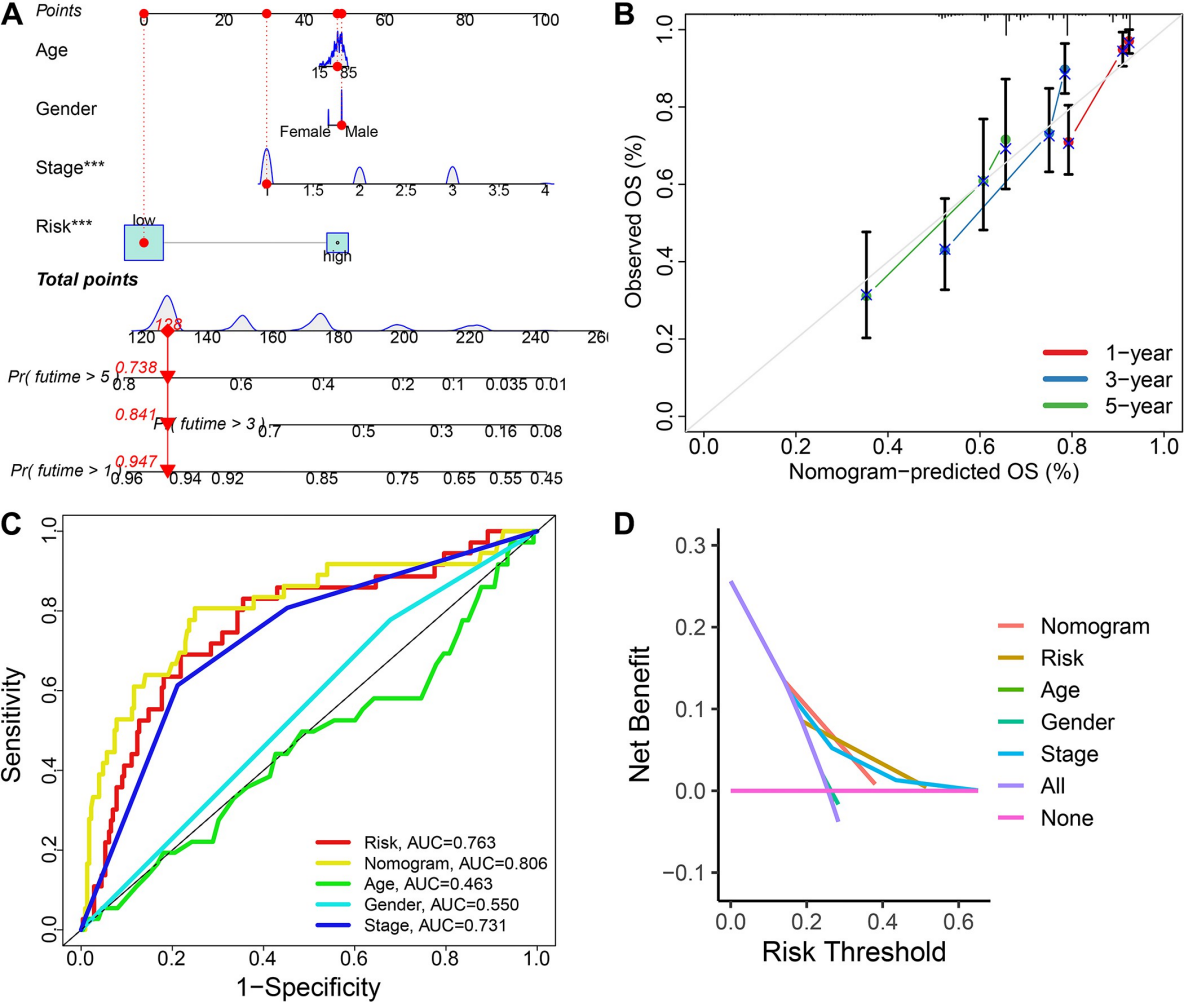
Construction of a nomogram based on M2-like macrophages-related prognostic signature (MRPS). (A) A predictive nomogram constructing with risk score, age, gender and clinical stage. (B) Calibration plots demonstrated that the actual 1-year, 3-year and 5-year survival times were highly consistent with the predicted survival times (C) ROC curve evaluated the performance of nomogram in predicting the clinical outcome of HCC patients. (D) DCA curve revealed that this nomogram had a high potential for clinical application.

As showed in Fig 4A and 4B, univariate and multivariate cox regression analysis indicated that MRPS-based risk score acted as an independent risk factor for the OS rate of HCC patients in TCGA, ICGC, GSE14520 and GSE76427 cohort. Compared with age, gender and clinical stage, the C-index of MRPS-based risk score was higher (Fig 4C–4F), suggesting the predictive value of risk score in predicting the OS rate of HCC patients was higher than age, gender and clinical stage. We also randomly collected 31 prognostic signatures that have developed for HCC (S3 Table) and calculated their C-index.We found that the C-index of MRPS was higher than that most of these 31 prognostic signatures, excepted ChenY signature, LiK signature, Yang signature and Yao signature (Fig 4G), indicating a better performance of our MRPS in predicting the OS rate of HCC than most of other prognostic signatures. As shown in Fig 5A, we then constructed a predictive nomogram to evaluate the mortality risk at 1, 3 and 5 years of HCC patients using age, sex, clinical stage and TRPS-based risk score. The calibration curves demonstrated the relative well conformity between speculated outcomes and observed outcomes (Fig 5B). Similar results were obtained from ICGC (S1A Fig), GSE14520 (S1B Fig) and GSE76427 (S1C Fig) cohort. Compared with risk score, age, gender, and clinical stage, the predictive value of nomogram in predicting the mortality risk at 1, 3 and 5 years of HCC patients, with an AUC of 0.806 in the ROC curve (Fig 5C). The decision curve analysis suggested a higher benefit of the nomogram compared with other clinical characteristics in predicting the clinical outcome of HCC patients (Fig 5D).

### MRPS-based distinct ecosystem in HCC

**Fig 6:**
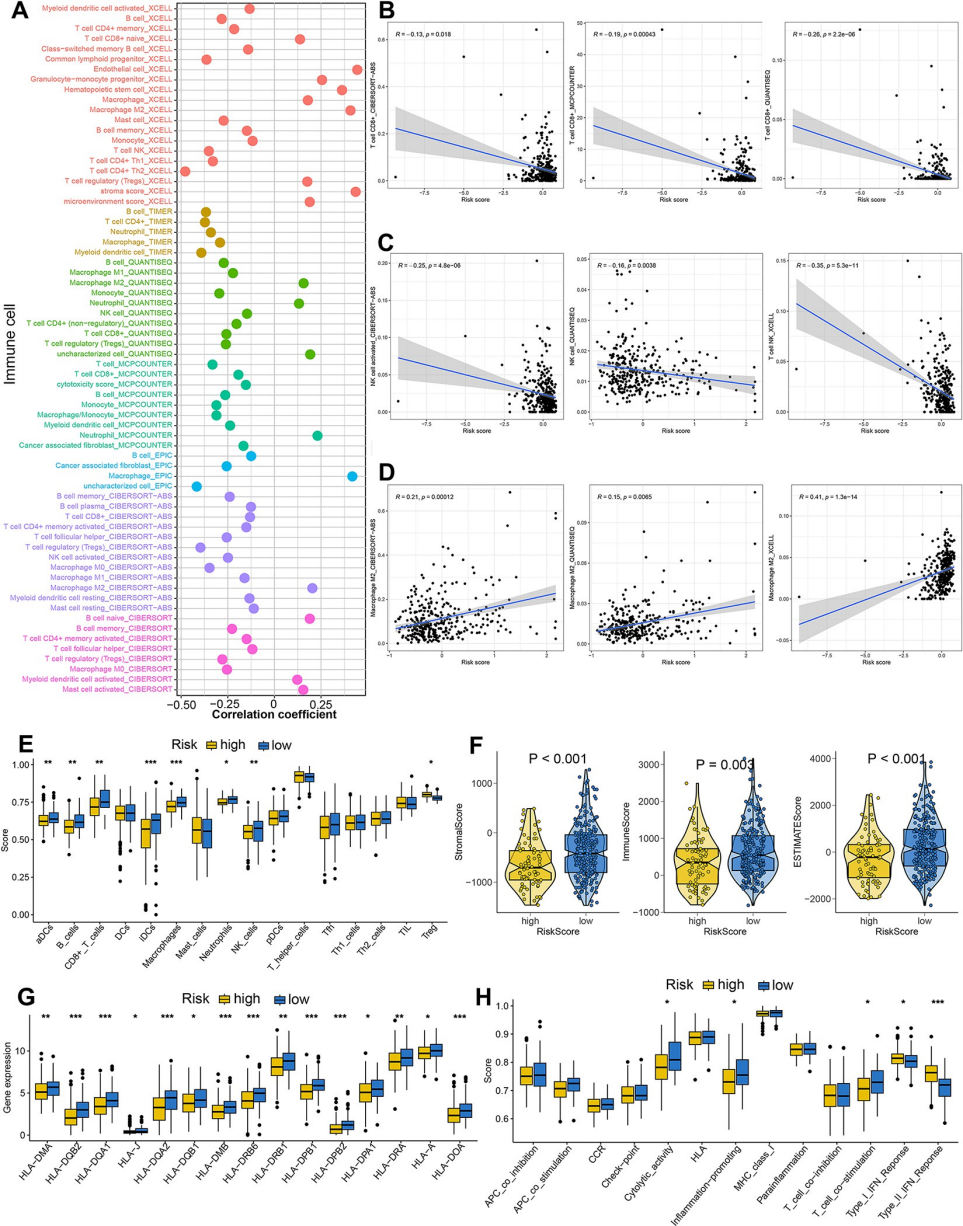
Immune microenvironment landscape in HCC patients with different risk score. (A) The correlation between risk score and the immune cell infiltration based on several state-of-the-art algorithms. Risk score showed negative correlation with the abundance of CD8^*+*^ T cells (B), NK cells (C) and positive correlation with the abundance of M2-like macrophage (D). (E) The level of immune cells in HCC patients with different risk score based on ssGSEA analysis. (F) Low risk score indicated a higher stromal score, immune score, and ESTIMAE score. (G-H) The score of HLA-related genes and immune-related functions in HCC patients with different risk score. *p<0.05, **p<0.01, ***p<0.001.

**Fig 7:**
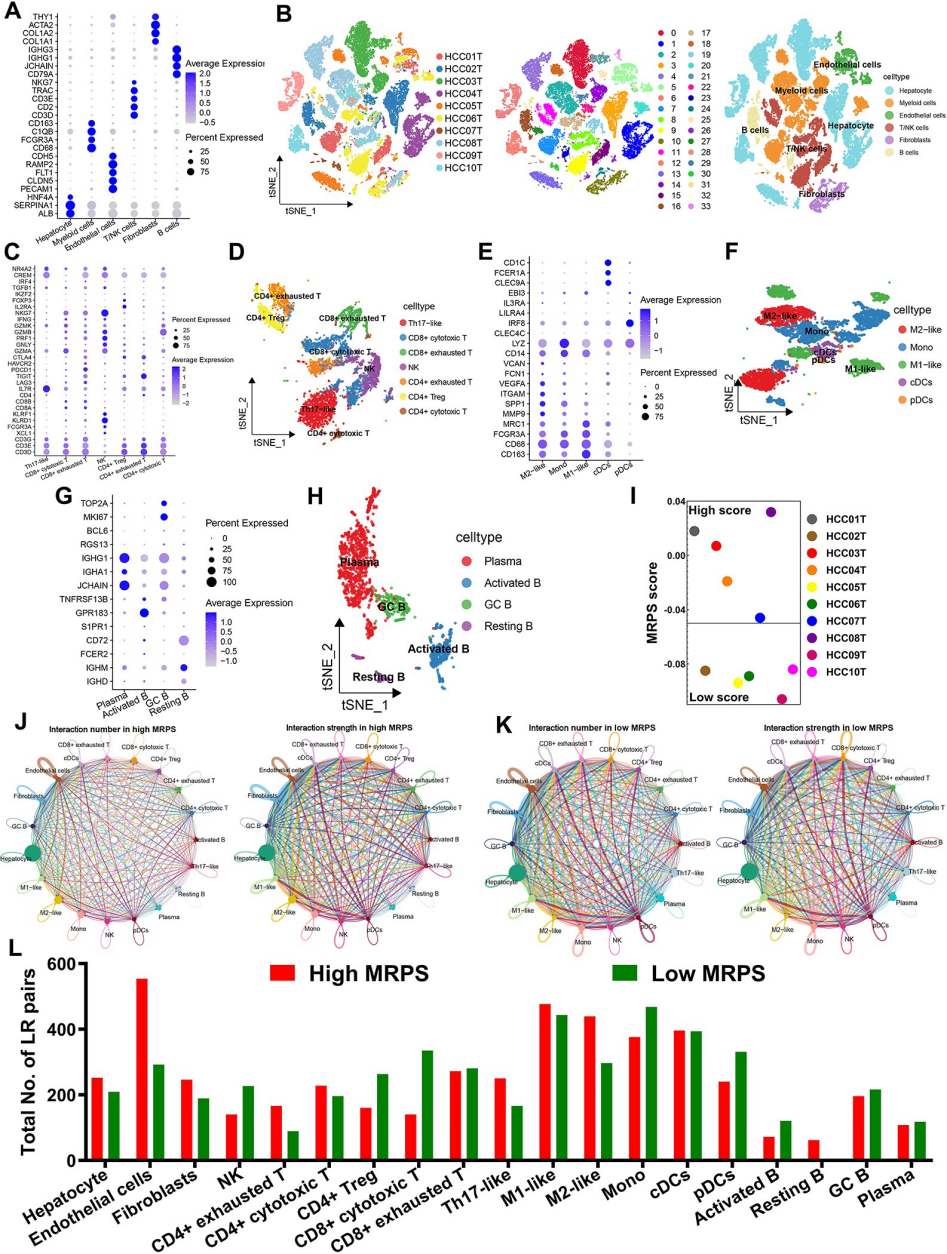
The distinct ecosystem in HCC patients with different MRPS score based on single cell analysis. (A) Dotplot showing the percentage of expressed cells and average expression levels of cell marker genes of major cell types. (B) T-distributed Stochastic Neighbor Embedding plot of 34 cell clusters and 6 major cell types from 10 HCC samples. (C-D) Based on expression pattern of cell markers, T/NK cells could be re-clustered into 7 cell types. (E-F) Myeloid cells could be clustered into M1-like macrophages, M2-like macrophages, monocyte (mono), plasmacytoid DCs (pDCs), and conventional dendritic cells (cDCs) according to the expression pattern of cell markers. (G-H) B cells were subdivided into plasma, GC B, resting B, and activated B according to the expression pattern of cell markers. (I) the MRPS score of 10 HCC patients. (J-K) CellChat revealing cell–cell communication network via known ligand-receptor pairs in HCC samples with different MRPS score. (L) Low MRPS-derived NK, CD8^+^ cytotoxic T, activated B possessed a higher number of ligand-receptor pairs, whereas the CD4^+^ exhauster T, Th17-like and M2-like macrophage possessed fewer pairs.

As shown in Fig 6A, significant correlation was obtained between risk score and immune cells. More specifically, MRPS-based risk score was negatively correlated with the abundance of immunoactivated cells, including CD8^+^ T cells and NK cells (Fig 6B and 6C). Significant correlation was obtained between MRPS-based risk score and immunosuppressive cells, including macrophage M2 (Fig 6D, *p*<0.05). Similarly, ssGSEA analysis revealed that HCC patients with low risk score had a higher abundance of immunoactivated cells, including aDCs, iDCs, B cells, CD8^+^ T cells and NK cells, and a lower abundance of immunosuppressive cells, including Treg (Fig 6E). Low risk score indicated a higher stromal score, immune score and ESTIMAE score in HCC (Fig 6F, all p<0.05). Further analysis revealed that the level of most of the HLA-related genes were higher in HCC patients with low risk score (Fig 6G, p<0.05). Moreover, low risk score indicated a higher cytolytic score, inflammation promoting score and T cell co-stimulation score in HCC (Fig 6H). These evidences revealed that the immune environment in HCC patients with low and high risk score is significantly distinct. In order to further verify our results, we then analyzed the ecosystem between HCC patients with different MRPS score. Based on the expression of cell markers, 10 HCC samples could be split into 34 clusters, which included 6 types of cells hepatocyte, myeloid cells, endothelial cells, T/NK cells, Fibroblasts, and B cells (Fig 7A and 7B). Based on expression pattern of cell markers, T/NK cells could be re-clustered into Th17-like, CD8+ cytotoxic T, CD8^+^ exhausted T, NK, CD4^+^ cytotoxic T, CD4^+^ exhausted T and Treg (Fig 7C and 7D). And myeloid cells could be clustered into M1-like macrophages, M2-like macrophages, monocyte (mono), plasmacytoid DCs (pDCs), and conventional dendritic cells (cDCs) according to the expression pattern of cell markers (Fig 7E and 7F). Similarly, B cells were subdivided into plasma, GC B, resting B, and activated B according to the expression pattern of cell markers (Fig 7G and 7H). These 10 HCC samples could be divided into high and low MRPS score groups using the median value of MRPS score as the cut-off (Fig 7I). To depict the differences in molecular interaction between the cells derived from the high MRPS score microenvironment and those from the low MRPS score microenvironment, we applied CellChat to construct a cell–cell communication network via known ligand-receptor pairs within these cells in HCC samples. The cell interaction number and strength in high and low MRPS score environment were showed in Fig 7J–7K. Notably, the low MRPS-derived NK, CD8^+^ cytotoxic T, activated B possessed a higher number of ligand-receptor pairs, whereas the CD4^+^ exhauster T, Th17-like and M2-like macrophage possessed fewer pairs (Fig 7L).

### MRPS-related risk score-based treatment strategy for HCC

**Fig 8:**
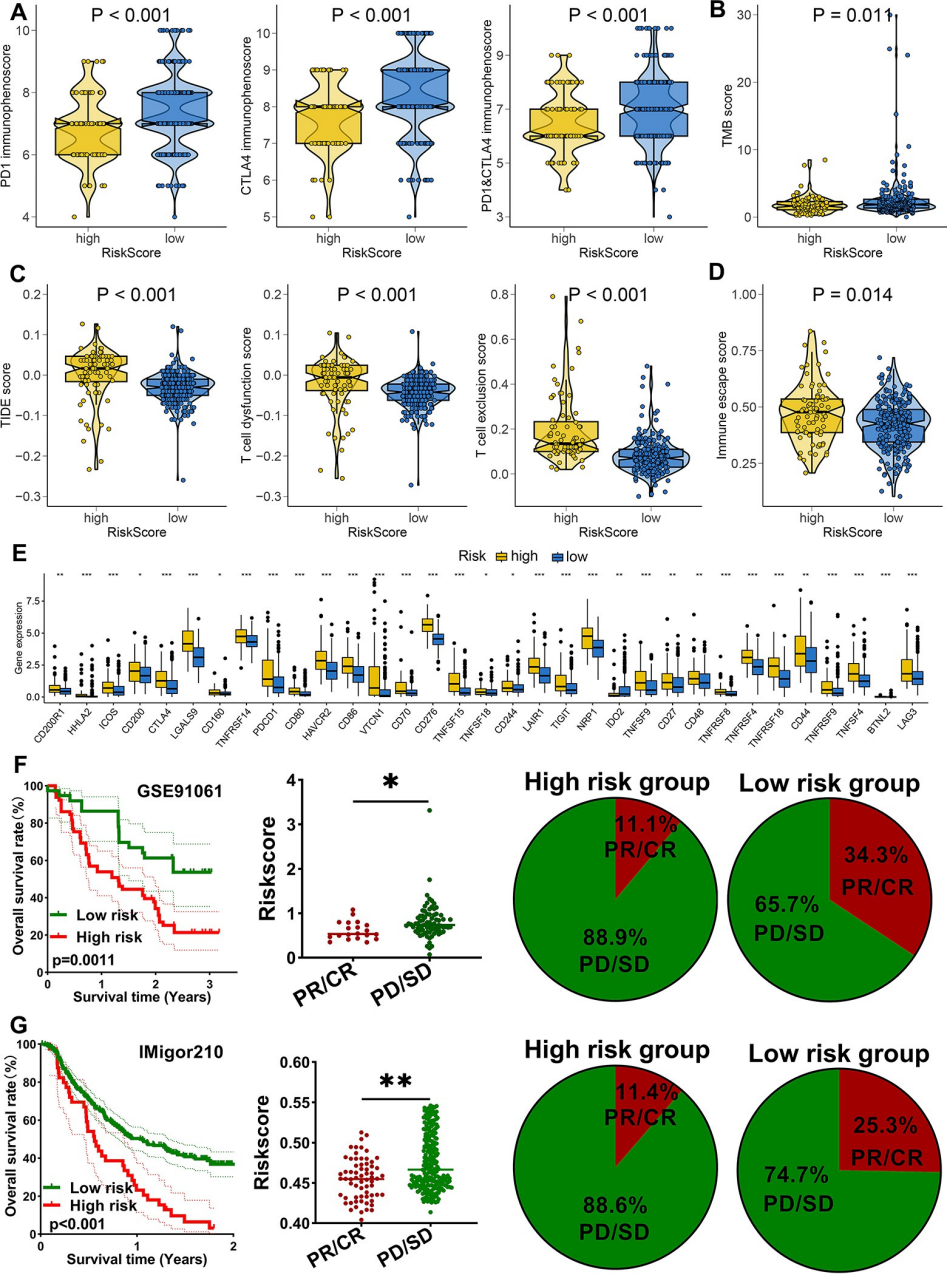
M2-like macrophages-related prognostic signature as an indicator for immunotherapy response in HCC. (A-B) The immunophenoscore and TMB score in HCC patients with high and low risk score. (C-D) The TIDE, T cell dysfunction score, T cell exclusion score and immune escape score in HCC patients with high and low risk score. (E) The level of common immune checkpoints in HCC patients with high and low risk score. The overall rate and immunotherapy response rate in patients with high and low risk score in GSE91061 (F) and IMvigor210 (G) cohort. *p<0.05, **p<0.01, ***p<0.001.

**Fig 9:**
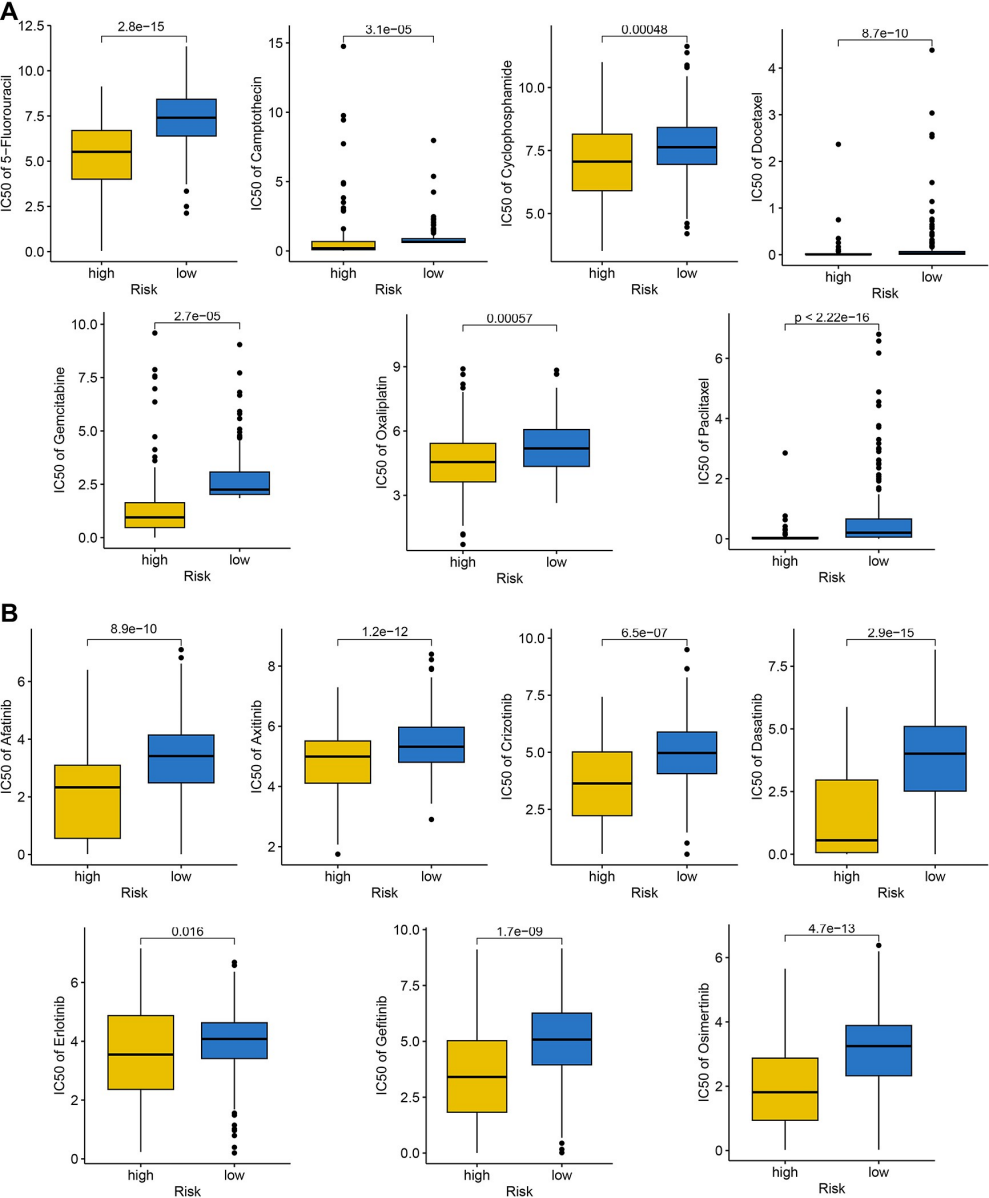
The IC50 value of common drugs for chemotherapy and targeted therapy. (A) High risk score indicated a lower IC50 value of 5-fluorouracil, camptothecin, cyclophosphamide, docetaxel, gemcitabine, oxaliplatin, and paclitaxel in HCC. (B) HCC patients with high risk score had a lower IC50 value of afatinib, axitinib, crizotinib, dasatinib, erlotinib, gefitinib, and Osimertinib.

We have it confirmed the significant distinct ecosystem in HCC patients with different risk score above and low risk score may be a related “hot” tumor phenotype. This, the immunotherapy response of patients with different risk score may be different. We then applied several approaches to evaluate the predictive value of MRPS-related risk score in immunotherapy response. As shown in Fig 8A, low risk score indicated a higher PD1 immunophenoscore, CTLA4 immunophenoscore, and PD1&CTLA4 immunophenoscore in HCC (^p^<0.05). We found that HCC patients with low risk score had a higher TMB score (Fig 8B). Compared with high risk score group, low risk score group had a lower TIDE score, T cell dysfunction score, T cell exclusion score (Fig 8C, all *p*<0.05). Moreover, low risk score indicated a lower immune escape score in HCC (Fig 8D, *p* = 0.014). As immune checkpoints played a vital role in inhibiting immune responses and promoted self-tolerance in cancer, we also explored the expression of immune checkpoints in HCC patients with different risk score. As expected, the level of most of immune checkpoints was lower in HCC patients with low risk score (Fig 8E, all *p*<0.05). Therefore, low risk score group may indicated a better sensitivity to immunotherapy. To furth verify our result, we then applied two immunotherapy cohorts. In GSE91061 cohort, patients with high risk score had a poor OS rate and the risk score in non-responders was significantly higher (Fig 8F, *p*<0.05). Moreover, the response rate in low risk score group was significant increased compared with high risk score group (Fig 8F). Similar results were obtained in IMigor210 cohort (Fig 8G). Chemotherapy and targeted therapy played a vital role in the therapy of HCC. We then explored the IC50 value of common drugs for chemotherapy and targeted therapy between high and low risk score groups. As shown in Fig 9A, high risk score indicated a lower IC50 value of 5-fluorouracil, camptothecin, cyclophosphamide, docetaxel, gemcitabine, oxaliplatin, and paclitaxel in HCC (all p<0.05). Moreover, HCC patients with high risk score had a lower IC50 value of afatinib, axitinib, crizotinib, dasatinib, erlotinib, gefitinib, and osimertinib (Fig 9B, all *p*<0.05). Thus, HCC with high risk score may have a better sensitivity to chemotherapy and targeted therapy.

### The distinct difference in cancer related hallmarks in different MRPS-based risk score group

**Fig 10:**
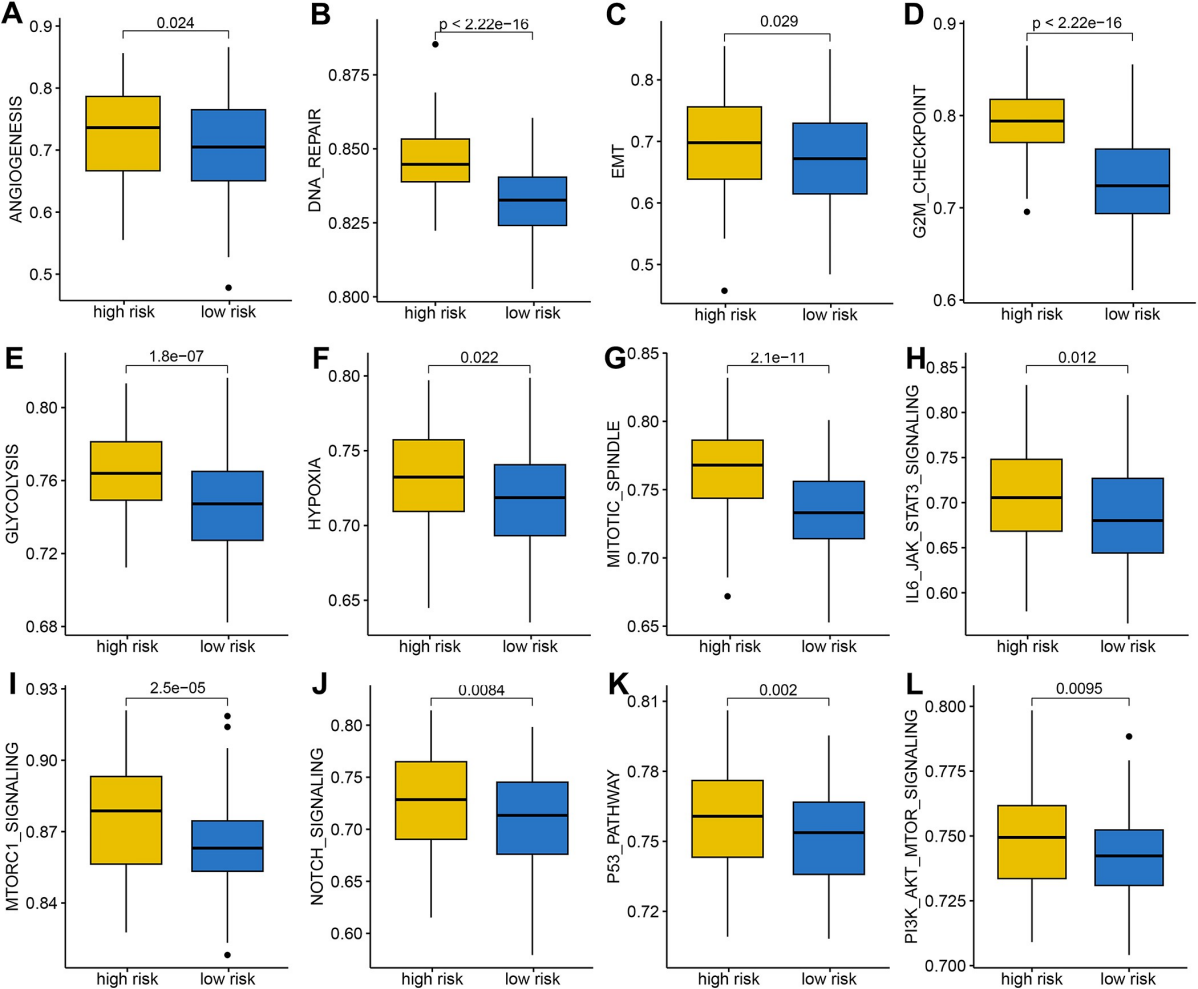
Gene set enrichment analysis of M2-like macrophages-related prognostic signature. The gene set score correlated with angiogenesis (A), DNA repair (B), EMT (C), G2M checkpoint (D), Glycolysis (E), Hypoxia score (F), Mitotic spindle (G), IL6-JAK-STAT3 signaling(H), MTOTC1 signaling (I), NOTCH signaling (J), P53 pathway score(K), and PI3K-ATK-MTOR signaling (L) in HCC patients with different risk score.

Because of the intense relationship between MRPS and prognosis, immune microenvironment, and immunotherapy response of HCC, we aim to apply gene set enrichment analysis to explore their biological process and internal connection. Interestingly, HCC patients with high risk score had a higher gene set score correlated with angiogenesis, DNA repair, EMT, G2M checkpoint, glycolysis, hypoxia, mitotic spindle, IL6-JAK-STAT3 signaling, MTORC1, NOTCH signaling, P53 pathway, and P13K-AKT-mTOR signaling (Fig 10A–10L), indicating that the activation of these biological processes may play a vital role in HCC tumorigenesis and progression.

## 4. Discussion

The present study developed a MRPS with 10 integrative machine learning algorithms and verified its predictivity in TCGA, ICGC and GEO cohorts. The optimal MRPS constructed by the combination of stepCox + superPC algorithm served as an independent risk factor and showed stable and powerful performance in predicting the overall survival rate of HCC patients with 2-, 3-, and 4-year AUCs of 0. 763, 0.751, and 0.699 in TCGA cohort. We also investigated the correlation between the MRPS and specific pathways, immune cell infiltration, and immune cell interactions in HCC.

In our study, we constructed a signature consisting of 12 MRGs (SNRPA, RECQL4, TRIM28, ERCC2, GRWD1, REEP4, PRMT1, PAF1, LAPTM4B, RANBP1, BCAT1, and PTK) which was a promising predictor of the prognosis of HCC. SNRPA was associated with microvascular invasion and promoted tumor metastasis in HCC [22]. Previous study also showed that high RECQL4 expression indicated a poor prognosis in HCC [23]. PRMT1 could accelerate tumor progression and metastasis via STAT3 signaling pathway in HCC [23]. Moreover, BCAT1 acted as a prognostic biomarker for HCC and promoted tumor progression via the AKT signaling pathway and EMT [24].

Actually, many macrophage-related prognostic signatures had been developed for other types of cancers. In bladder cancer, a six-gene prognostic signature associated macrophage was developed [25]. Moreover, macrophage phenotypic-related signature could indicate the prognosis of pancreatic cancer patients [26]. Qianxia *et al*. also constructed a tumor-associated macrophage related signature for the prognosis of ovarian cancer patients [27].

Immunotherapy has revolutionized the situation of patients with unresectable cancers [28]. Though many drug for blocking the immune checkpoints have been approved for the immunotherapy of cancers, including HCC, limited effective biomarkers could accurately predict immunotherapy efficacy. Considering the significant correlation between M2-like macrophage and immune infiltration, we then explore the role of MRPS in predicting the immunotherapy response of patients. An increased TIDE score indicates a greater likelihood of immune escape and less effectiveness of ICI treatment [29]. Our result suggested that low risk score indicated a lower TIDE score in HCC. TMB acted as an indicator for immunotherapy and higher TMB indicated a better response to immunotherapy [30]. The results showed that HCC patients with low risk score had a higher TMB score and lower tumor escape score. IPS, primarily developed from TCGA RNA-seq data, was designed to predict patient responses to immune checkpoint inhibitor treatments [31]. It is confirmed that low-risk populations respond more effectively to immunotherapy, as evidenced by the higher IPS, which is consistent with our findings. Thus, low MRPS-based risk score may indicate a batter response to immunotherapy in HCC.

To clarify why the prognosis, immune microenvironment, and immunotherapy response of HCC with different risk score is distinct, we then used gene set enrichment analysis to explore their biological process and internal connection. As a result, HCC patients with high risk score had a higher gene set score correlated with angiogenesis, DNA repair, EMT, G2M checkpoint, glycolysis, hypoxia, mitotic spindle, IL6-JAK-STAT3 signaling, MTORC1, NOTCH signaling, P53 pathway, and P13K-AKT-mTOR signaling. The activation of these cancer-related hallmarks may be more pronounced in HCC patients with high risk score. Actually, macrophages could stimulate angiogenesis, enhance tumor cell migration and invasion in HCC [32]. Another study showed that macrophage-Associated PGK1 phosphorylation could promotes aerobic glycolysis and tumorigenesis [33]. Previous study showed that the Notch signaling pathway regulated macrophage polarization in HCC [34].

## 5. Conclusion

Our study proposed a novel MRPS using 10 integrative machine learning algorithms. This MRPS acted as a risk factor for the prognosis of HCC patients and it could predict the ecosystem and immunotherapy response of HCC patients.

## 6. Ethics approval and consent to participate

This study did not involve human participants or human material. The transcriptome data and clinical information were downloaded from the TCGA and GEO databases, which are publicly available. Therefore, this study did not require the approval of the local ethics committee. All methods were performed in accordance with the Declaration of Helsinki and relevant regulations.

## Supporting information

S1 Fig Predicting nomogram based on M2-like macrophages-related prognostic signature (MRPS). A predictive nomogram and calibration plots in ICGC (A), GSE14520 (B) and GSE76427 (C) cohort.
(JPG)

S1 Table Macrophage M2 related markers identified by WGCNA analysis. (XLSX)

S2 Table Differentially expressed Macrophage M2 related markers in HCC. (XLSX)

S3 Table Prognostic signatures that have developed for HCC. (XLSX)
