# Supplementary figures and images for: Identification of M2-like macrophage-related signature for predicting the prognosis, ecosystem and immunotherapy response in hepatocellular carcinoma
Source: PLoS One. 2023 Sep 19;18(9):e0291645. doi: 10.1371/journal.pone.0291645 (PMC10508629; doi:10.1371/journal.pone.0291645)

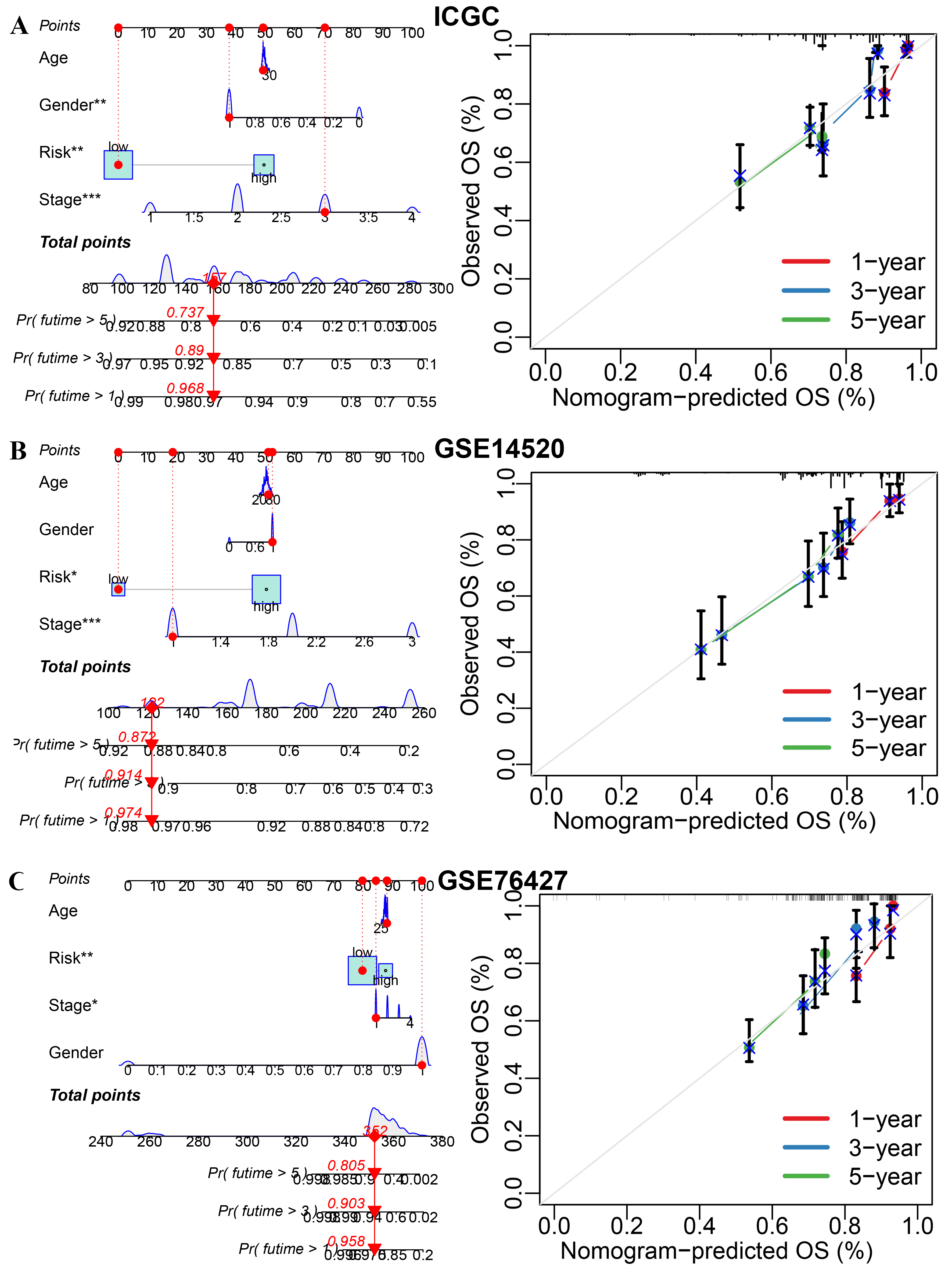

Supplement: S1 Fig — A predictive nomogram and calibration plots in ICGC (A), GSE14520 (B) and GSE76427 (C) cohort. (JPG) [file pone.0291645.s001.jpg]
